# Vitamin D and Calcium Addition during Denosumab Therapy over a Period of Four Years Significantly Improves Lumbar Bone Mineral Density in Japanese Osteoporosis Patients

**DOI:** 10.3390/nu10030272

**Published:** 2018-02-27

**Authors:** Takako Suzuki, Yukio Nakamura, Hiroyuki Kato

**Affiliations:** 1 Department of Orthopaedic Surgery, Shinshu University School of Medicine, Asahi 3-1-1, Matsumoto 390-8621, Japan; takako1119@shinshu-u.ac.jp (T.S.); hirokato@shinshu-u.ac.jp (H.K.); 2 Department of Orthopedic Surgery, Showa-Inan General Hospital, Akaho 3230, Komagane 399-4117, Japan

**Keywords:** bone mineral density, bone turnover markers, denosumab, primary osteoporosis, vitamin D

## Abstract

This study investigated whether or not vitamin D and calcium supplementation affected bone metabolism and bone mineral density (BMD) over a period of four years of denosumab therapy in patients with primary osteoporosis. Patients were divided into a denosumab monotherapy group (22 cases) or a denosumab plus vitamin D and calcium supplementation group (combination group, 21 cases). We measured serum bone alkaline phosphatase (BAP), tartrate-resistant acid phosphatase (TRACP)-5b, urinary N-terminal telopeptide of type-I collagen (NTX), and BMD of the lumbar 1–4 vertebrae (L-BMD) and bilateral hips (H-BMD) at baseline and at 12, 24, 36, and 48 months of treatment. There were no significant differences in patient background. Serum BAP, TRACP-5b, and urinary NTX were significantly and comparably inhibited in both groups from 12 to 48 months versus baseline values. L-BMD was significantly increased at every time point in both groups, while H-BMD was significantly increased at every time point in the combination group only. There were significant differences between the groups for L-BMD at 24, 36, and 48 months (*P* < 0.05) and for H-BMD at 12 months (*P* < 0.05). Compared with denosumab monotherapy, combination therapy of denosumab plus vitamin D and calcium significantly increased H-BMD at 12 months and L-BMD from 24 to 48 months. These findings indicate that continuous vitamin D and calcium supplementation is important, especially for 12 months to improve H-BMD and from 24 to 48 months to improve L-BMD.

## 1. Introduction

Osteoporosis is the most common bone disease and affects millions of people worldwide, particularly the elderly and post-menopausal women. Thus, appropriate lifestyle, nutritional supplement, and pharmacological therapeutic choices are required. 

The American Association of Clinical Endocrinologists (AACE)/American College of Endocrinology Medical Guidelines for Practice very recently declared alendronate, risedronate, zoledronic acid, and denosumab as suitable initial therapies for most osteoporosis patients, especially those at high risk of fracture [1]. 

Denosumab is a fully human monoclonal antibody against receptor activator of nuclear factor-kappa B ligand (RANKL) that has been shown to selectively inhibit osteoclastogenesis. Consequently, denosumab abrogates bone resorption, increases bone mineral density (BMD), and prevents fragility fractures [2,3]. We and others have reported that the drug is a good therapeutic option for primary as well as secondary osteoporosis and fracture prevention in young and elderly patients [4,5,6,7,8,9,10,11,12,13]. However, most reports focus on the short-term efficacy of denosumab, i.e., 1 or 2 years, and its efficacy in real-world settings is largely unknown. 

Known as calcitriol, active vitamin D (1,25-dihidroxycholecalciferol; 1,25(OH)_2_D_3_) regulates calcium metabolism [14]. Native vitamin D (from nutrition) is cholecalciferol. The latter is hydroxylated in the liver to become 25(OH)D_3_ (calcifediol), which is further hydroxylated in the kidneys to form 1,25(OH)_2_D_3_ [15]. Soon after the approval of denosumab use in Japan, calcium and vitamin D supplementation has been advocated to prevent hypocalcemia in osteoporosis treatment using denosumab, such as with the newly developed Denotas Chewable^®^ (vitamin D and calcium supplementation tablets).

In our previous study [9], vitamin D and calcium supplementation was required with denosumab for primary osteoporosis since they improved bone metabolism and BMD to a greater extent after 1 year. To the best of our knowledge, however, there have been no reports on the long-term differences in bone metabolism and BMD between denosumab monotherapy and combination therapy with vitamin D and calcium in the clinical setting. 

It is unclear if active vitamin D provides additive BMD and bone turnover marker gains in patients undergoing long-term bisphosphonate (BP) usage. We recently found that the addition of eldecalcitol with BP substantially increased BMD over time [16]. On the other hand, we have also observed that serum 25(OH)D_3_ concentrations are significantly increased after 3 years of BP therapy, even without vitamin D supplementation [17]. Thus, additional vitamin D may not be required in long-term BP therapy for osteoporosis, but the comparative effects of extended denosumab treatment with or without vitamin D and calcium supplementation on bone metabolism and BMD remain uncertain. 

In this study, we examined the 1) efficacy of denosumab therapy, and 2) the transitions in serum calcium, phosphorus, 1,25(OH)_2_D_3_, and parathyroid hormone (PTH), which plays important roles in determining bone resorption and bone mass, with or without vitamin D and calcium supplementation over a period of four years in primary Japanese osteoporosis patients.

## 2. Patients and Methods

Forty-three patients were recruited at Shinshu University School of Medicine and Showa-Inan General Hospital between 2013 and 2017 (Table 1). The inclusion criteria for this investigation were primary treatment-naïve osteoporotic patients with low (T-score less than −2.5 SD) bilateral hip BMD (H-BMD). The exclusion criteria were chronic renal failure (an estimated glomerular filtration rate of < 40 mL/min/1.73 m^2^) with bone metabolic disorder or diabetes mellitus possibly affecting osteoporosis. Patients were divided into two groups: 22 receiving denosumab alone (denosumab monotherapy group) and 21 receiving denosumab and vitamin D supplementation (combination group) (Table 1). All patients were diagnosed as having primary osteoporosis. No patient had been pretreated with any BP before denosumab treatment (Table 1). The diagnosis of primary osteoporosis was made in accordance with the revised criteria established by the Japanese Society of Bone and Mineral Research [18]. All patients received denosumab (60 mg, s.c.) once every 6 months. Subjects in the combination group were given newly approved vitamin D and calcium supplementation tablets (610 mg of calcium, 400 IU of cholecalciferol, 30 mg of magnesium) daily during the denosumab regime. 

The percent changes in serum concentrations of calcium, phosphorus, whole PTH, 1,25(OH)_2_D_3_, bone alkaline phosphatase (BAP), tartrate-resistant acid phosphatase (TRACP)-5b, and urinary N-terminal telopeptide of type-I collagen (NTX) were measured at baseline and at 12, 24, 36, and 48 months of treatment. BAP was measured as a bone-formation marker using a chemiluminescent enzyme immunoassay. TRACP-5b and urinary NTX (Osteomark®; Ostex International, Seattle, WA, USA) were assessed as markers of bone resorption using an enzyme-linked immunosorbent assay. Whole PTH and 1,25(OH)_2_D_3_ were measured by immunoradiometric assays. After an overnight fast, serum and first-void urine samples were collected between 08:30 and 10:00 a.m. Immunoassays were carried out by SRL (Tokyo, Japan). 

The percent changes in BMD were calculated using a dual-energy X-ray absorption fan-beam bone densitometer (Lunar Prodigy; GE Healthcare, Waukesha, WI, USA) at the lumbar 1–4 vertebrae of the posteroanterior spine (L-BMD) and the bilateral hips. 

Results are expressed as the mean ± standard error of the mean. For both groups, we compared the changes in serum calcium, phosphorus, whole PTH, 1,25(OH)_2_D_3_, bone turnover markers, L-BMD, and H-BMD with baseline values at each time point using the Bonferroni correction method for multiple comparisons. Comparisons of the above parameters between the two groups at each measurement point were performed using Welch’s *t*-test. Differences were considered statistically significant at *P* < 0.05. 

The study protocol was approved by the Ethics Committee of Shinshu University School of Medicine (Matsumoto, Japan) and Showa-Inan General Hospital (Komagane, Japan). This investigation was carried out in accordance with the ethical standards set forth in the Declaration of Helsinki (2014 revision). Written informed consent was obtained from all patients.

## 3. Results

There were no significant differences in patient background between the denosumab monotherapy group and combination group (Table 1). No serious adverse events, such as hypocalcemia or atypical fracture, occurred during the study. 

### 3.1. Serum Albumin-Corrected Concentrations of Calcium, Phosphorus, Whole PTH, and 1,25(OH)_2_D_3_

**Figure 1:**
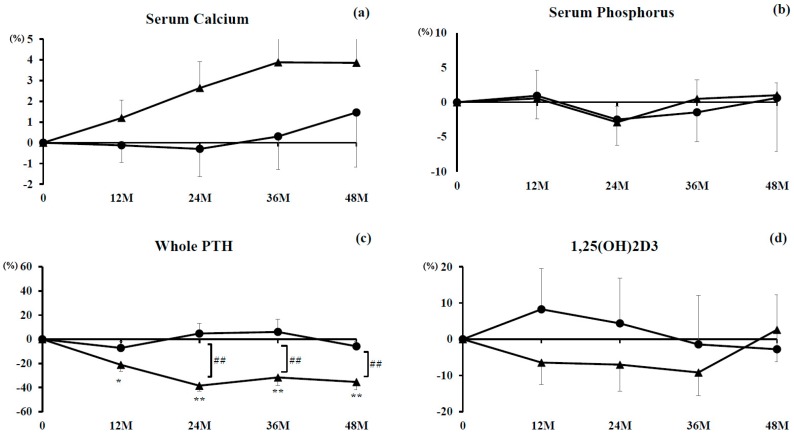
Percent changes in concentrations of serum albumin-corrected calcium (**a**); phosphorus (**b**); whole parathyroid hormone (PTH) (**c**); and 1,25(OH)_2_D_3_ (**d**). Closed circles indicate the denosumab monotherapy group and closed triangles indicate the combination group. Single (*P* < 0.05) and double (*P* < 0.01) asterisks denote significant differences compared with pretreatment values. Double hashtags denote a significant difference (*P* < 0.01) between the denosumab monotherapy and combination groups at indicated time points.

No significant differences in serum calcium concentration were found in either group with their respective baseline or between the groups at any time point (Figure 1a). Percent changes hovered around baseline in the denosumab monotherapy group and were slightly increased in the combination group throughout the study period. 

Similarly for percent changes in serum phosphorus, no significant differences were seen for either group and their respective baseline or between the groups at any time point (Figure 1b). Percent changes transitioned comparably in both groups during the observational period. 

The percent changes in serum concentrations of the whole PTH were significantly inhibited at 12 months (*P* < 0.05) and at 24, 36, and 48 months (*P* < 0.01) in the combination group. However, they remained around baseline in the denosumab monotherapy group. Significant differences were noted at 24, 36, and 48 months between the groups (*P* < 0.01) (Figure 1c).

No significant differences were seen for percent changes in serum 1,25(OH)_2_D_3_ for either group and their respective baseline or between the groups at any time point (Figure 1d). 

### 3.2. Markers of Bone Turnover

#### 3.2.1. Marker of Bone Formation

**Figure 2:**
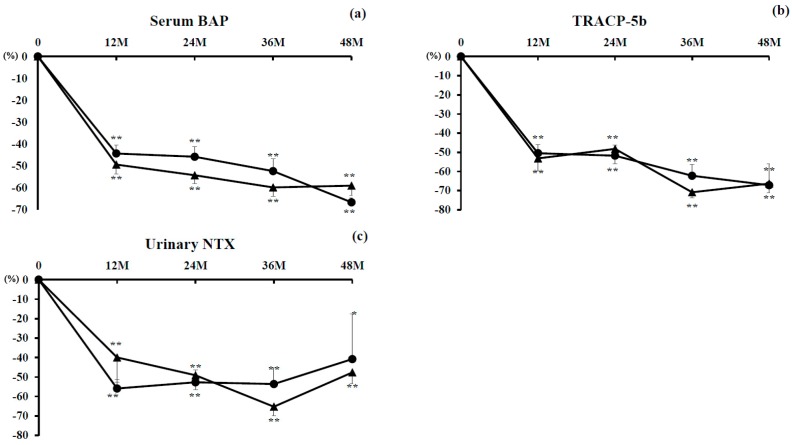
Percent changes in concentrations of bone alkaline phosphatase (BAP) (**a**) and tartrate-resistant acid phosphatase (TRACP)-5b (**b**) and of urinary N-terminal telopeptide of type-I collagen (NTX) (**c**). Closed circles indicate the denosumab monotherapy group and closed triangles indicate the combination group. Single (*P* < 0.05) and double (*P* < 0.01) asterisks denote significant differences compared with pretreatment values.

Percent changes in serum concentrations of BAP were significantly decreased from 12 to 48 months in both groups (*P* < 0.01). There were no significant differences between the groups (Figure 2a).

#### 3.2.2. Markers of Bone Resorption

Percent changes in serum concentrations of TRACP-5b were significantly suppressed from 12 to 48 months in both groups (*P* < 0.01). There were no significant differences between the groups (Figure 2b). 

Percent changes in concentrations of urinary NTX were also significantly suppressed from 12 to 48 months in both groups (*P* < 0.01, and *P* < 0.05 at 48 months only in the denosumab monotherapy group). There were no significant differences between the groups (Figure 2c).

#### 3.2.3. L-BMD and H-BMD

**Figure 3:**
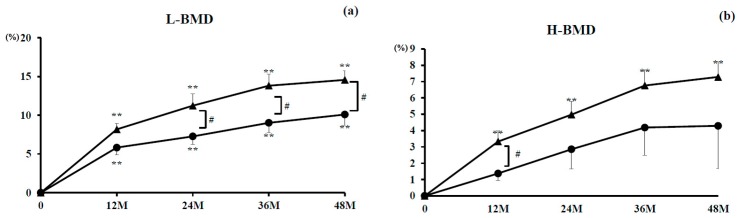
Percent changes in lumbar 1–4 spine bone mineral density (L-BMD) and total hip BMD (H-BMD). Closed circles indicate the denosumab monotherapy group and closed triangles indicate the combination group. Double asterisks denote a significant difference (*P* < 0.01) compared with pretreatment values. Single hashtag denotes a significant difference (*P* < 0.05) between the groups at indicated time points.

Percent changes in L-BMD were significantly increased throughout treatment in the denosumab monotherapy group (10.1% increase at 48 months) (all *P* < 0.01) and the combination group (14.6% increase at 48 months) (all *P* < 0.01) compared with baseline values. There were significant differences at 24, 36, and 48 months (*P* < 0.05) between the groups (Figure 3a). 

Percent changes in H-BMD were significantly increased throughout treatment in the combination group (7.3% increase at 48 months) (all *P* < 0.01) and substantially increased for 48 months in the denosumab monotherapy group (4.3% increase at 48 months). There was a significant difference at 12 months between the groups (*P* < 0.05) (Figure 3b).

## 4. Discussion

We report for the first time long-term comparative data for denosumab with or without vitamin D and calcium supplementation in Japanese patients with primary osteoporosis. Compared with denosumab monotherapy, combination therapy significantly inhibited percent changes in PTH and increased percent changes in H-BMD at 12 months and L-BMD from 24 to 48 months. 

Denosumab is a potent and effective anti-resorptive compound. We and others have reported a significantly decreased risk of vertebral fracture and absence of hypocalcemia with denosumab for 1 year [9,19]. However, studies on the effectiveness and/or adverse effects of denosumab with or without vitamin D supplementation in osteoporosis patients for longer than 3 years in a real-world setting are lacking. Body et al. observed that denosumab without calcium and vitamin D caused significant hypocalcemia [20], although we earlier showed no hypocalcemia or other serious adverse effects and no significant calcium metabolic change [9], which was consistent with the current data. These results indicate that hypocalcemia can be prevented during denosumab treatment by supplementation with vitamin D and calcium.

Ebina’s group and our own recently reported that denosumab plus active vitamin D combination therapy significantly increased femoral neck BMD values compared with denosumab plus native vitamin D [11,12]. In the present study, serum PTH concentrations were significantly decreased and serum calcium concentrations rose in the combination group over the four years. We earlier described significantly increased serum PTH and significantly decreased serum calcium in a denosumab monotherapy group [7]. Together, these findings suggest that, in the short-term, serum calcium concentrations decrease to thereby increase PTH concentration in denosumab monotherapy patients, while in the long-term, vitamin D and calcium addition increase calcium to decrease PTH. It was noteworthy that serum calcium and PTH concentrations did not change without vitamin D addition during denosumab therapy between 1 and 4 years. 

Prior studies have shown that active vitamin D administration decreased serum concentrations of PTH [7,21], which was consistent with the present data. Signaling of PTH receptors in osteoblasts and osteocytes can increase the ratio of RANKL/osteoprotegerin (a decoy receptor of RANKL) to increase the recruitment and activity of osteoclasts and stimulate bone resorption [21]. Thus, the inhibitory effects on PTH caused by vitamin D administration might have resulted in larger gains in BMD in the combination group than in the monotherapy group. 

In our cohort, denosumab alone increased L-BMD as much as 10.1% and H-BMD as much as 4.3% at 48 months, while denosumab plus vitamin D and calcium supplementation augmented L-BMD as much as 14.6% and H-BMD as much as 7.3% at 48 months. Moreover, there was a significant difference in H-BMD at 12 months in the combination group over the denosumab monotherapy group and more significant gains in L-BMD from 12 to 48 months in the combination group. Ebina et al. found that PTH values were significantly higher in a native vitamin D group than in an alfacalcidol group, which had shown increased BMD, without differences in bone turnover inhibitory effects between the groups [11]. Taken together, vitamin D addition may significantly increase BMD owing to a decrease in serum PTH. 

Heckman et al. described that, in elderly patients with osteoporosis who were unresponsive to BPs, vitamin D addition could improve BMD at the lumbar spine to consequently prevent fracture [22]. Our study showed that H-BMD at 12 months and L-BMD at 24, 36, and 48 months were significantly improved by the addition of vitamin D and calcium during denosumab treatment in primary osteoporosis in the absence of fractures during the observation period. The above evidence confirms that an increase in BMD reduces the risk of fracture and that combination therapy of denosumab with vitamin D may be optimal.

Lastly, Holick reported that serum 25(OH)D_3_ was the only barometer for vitamin D status in osteoporosis patients and that serum 1,25(OH)_2_D_3_ provided limited information about vitamin D status [23]. Since we did not examine serum 25(OH)D_3_ concentrations in this study, the precise vitamin D status was unknown in the combination group. 

The main limitations of this investigation were its relatively small sample size and that serum 25(OH)D_3_ status was not measured. Further studies are required to ascertain to what extent fractures can be prevented over longer periods. 

## 5. Conclusions

No adverse effects, such as atypical fracture or hypocalcemia, occurred in either the denosumab monotherapy group or the combination group. H-BMD at 12 months and L-BMD from 24 to 48 months were significantly improved in combination therapy of denosumab with vitamin D and calcium. Thus, it is highly recommended that these supplements are included with denosumab administration for primary osteoporosis carrying a high risk of hip and lumbar fracture.

## Figures and Tables

**Table 1:** Patient characteristics prior to denosumab treatment.

Characteristic	Denosumab (*n* = 22)	Combination (*n* = 21)	*p*-Value
Age (years)	72.3 ± 2.0	72.1 ± 2.7	0.94
Gender (F:M)	20:2	18:3	
BMI (kg/m^2^)	21.8 ± 0.9	21.5 ± 0.7	0.74
Serum-corrected calcium (mg/dL)	9.2 ± 0.1	9.1 ± 0.1	0.19
Serum phosphorus (mg/dL)	3.7 ± 0.1	3.5 ± 0.2	0.40
Serum BAP (μg/L)	21.4 ± 2.3	22.1 ± 2.5	0.85
TRACP-5b (mU/dL)	595.1 ± 45.7	593.4 ± 49.2	0.98
Urinary NTX (nmol BCE/mmol CRE)	52.6 ± 5.6	49.7 ± 5.6	0.67
1,25(OH)_2_D_3_ (pg/mL)	57.9 ± 4.7	57.6 ± 6.7	0.97
Serum whole PTH (pg/mL)	28.9 ± 2.8	30.0 ± 2.8	0.77
Lumbar 1–4 BMD (g/cm^2^)	0.789 ± 0.02	0.798 ± 0.04	0.84
Total hip BMD (g/cm^2^)	0.657 ± 0.02	0.672 ± 0.03	0.68

BMI: body mass index; BAP: bone specific alkaline phosphatase; TRACP-5b: tartrate-resistant acid phosphatase-5b; NTX: N-terminal telopeptide of type-I collagen; PTH: parathyroid hormone; BMD: bone mineral density. Results are the mean ± standard error of the mean. A *P*-value of < 0.05 was considered statistically significant.
